# Cannabinoids for Acute Pain Management: Approaches and Rationale

**DOI:** 10.1007/s11916-024-01252-4

**Published:** 2024-04-12

**Authors:** Mihai O. Botea, Lukas Andereggen, Richard D. Urman, Markus M. Luedi, Carolina S. Romero

**Affiliations:** 1 Department of Anaesthesiology and Critical Care, Medicover Pelican Clinic Hospital, Oradea, Romania; 2 Department of Neurosurgery, Cantonal Hospital of Aarau, Aarau, Switzerland; 3 Faculty of Medicine, University of Bern, Bern, Switzerland; 4 Department of Anaesthesiology, The Ohio State University, Columbus, OH 43210 USA; 5 Department of Anaesthesiology, Rescue- and Pain Medicine, Cantonal Hospital of St, Gallen, St. Gallen, Switzerland; 6 Department of Anaesthesiology and Pain Medicine, Inselspital, Bern University Hospital, University of Bern, Bern, Switzerland; 7 Department of Anaesthesiology and Critical Care, Hospital General Universitario De Valencia, Valencia, Spain; 8 Research Methods Department, Universidad Europea de Valencia, Valencia, Spain

**Keywords:** Acute pain management, Cannabinoids, Tetrahydrocannabinol, Cannabidiol

## Abstract

**Purpose of the Review:**

Acute pain management remains a challenge and postoperative pain is often undermanaged despite many available treatment options, also including cannabinoids.

**Recent Findings:**

In the light of the opioid epidemic, there has been growing interest in alternative care bundles for pain management, including cannabinoids as potential treatment to decrease opioid prescribing. Despite the lack of solid evidence on the efficacy of cannabinoids, their use among patients with pain, including those using opioids, is currently increasing. This use is supported by data suggesting that cannabinoids could potentially contribute to a better pain management and to a reduction in opioid doses while maintaining effective analgesia with minimum side effects.

**Summary:**

The scientific basis for supporting the use of cannabis is extensive, although it does not necessarily translate into relevant clinical outcomes. The use of cannabinoids in acute pain did not always consistently show statistically significant results in improving acute pain. Large randomized, controlled trials evaluating diverse cannabis extracts are needed in different clinical pain populations to determine safety and efficacy.

## Introduction

Acute pain is a common symptom of a wide variety of triggering conditions, and the primary reason most patients access healthcare systems [1•]. From the pain perspective, tension-type headache is the primary cause of morbidity, followed by musculoskeletal pain [2]. However, the increasing surgical requirements and the aging of the population make perioperative pain a challenge for clinicians and patients, being an undermanaged condition despite advances in pain management modalities [3].

Furthermore, the opioid crisis, especially in North America, has prompted a clear and present urgency to find alternative strategies to treat acute pain [4, 5]. In addition to traditional pain killers such as acetaminophen, non-steroidal anti-inflammatories, and gabapentinoids, novel alternatives or various adjuvants are gaining popularity [6], such as N-methyl-D-aspartate (NMDA) receptor antagonists, alpha-2adrenergic agonists, glucocorticoids, and selective serotonin reuptake inhibitors are increasingly used in the management of acute pain to spare or avoid opioids. Thus, in light of the opioid epidemic, there has been growing interest in opioid alternatives for pain management including cannabinoids, namely (THC) and cannabidiol (CBD), which are claimed to produce antinociceptive effects [7].

Despite the lack of solid evidence on the efficacy of cannabinoids for the management of pain, their use among patients with pain, including those using opioids, is currently increasing [8]. This use is motivated by data suggesting that cannabinoids could potentially contribute to reducing the doses of opioids while maintaining effective analgesia [9•].

In fact, the global use of cannabinoids for both medical purposes and recreational is accelerating at an unprecedented pace. There is a growing trend in legalization of cannabis globally, thus cannabinoids have been gaining mainstream acceptance for potential efficacy in pain management. However, there are some unanswered questions about the evidence backing up the use of cannabinoids in pain management. In the United States (US), cannabis has received a statute as a therapeutic option for cancer pain without scientific rigor or regulatory oversight needed for other traditional therapies. This widespread use of cannabis products and the lack of education among clinicians and patients alike have triggered the American Society of Regional Anesthesia and Pain Medicine to create and publish in 2022 a practice guideline on the management of the perioperative patient on cannabis and cannabinoids in order to facilitate decision-making [10••].

By 2030, the cannabinoids market is estimated to be 100 billion US dollars, which will far exceed that of the largest pharmaceutical company in the world. This fast-growing market is also due to legalization interests and to the immense positive media attention, which might shape the magnitude of placebo responses in future trials. As the push for legalization grows, we consider that it is crucial for the perioperative clinicians to be aware of the implications of increasing cannabinoid levels in the body and their potential (or lack of) therapeutic benefits.

## The Endocannabinoid System

There are three known types of cannabinoids: (1) the phytocannabinoids extracted from the cannabis plant, (2) synthetic cannabinoids (e.g., ajulemic acid, nabilone) based upon the chemical structure of THC or other ligands, and (3) the endogenous cannabinoids or endocannabinoids (eCBs). Endocannabinoids are natural chemicals such as anandamide (ANA or AEA, N-arachidonoylethanolamine) and 2-arachidonoylglycerol (2-AG) found in animals whose basic functions are “relax, eat, sleep, forget, and protect” [11].

They studied and recognized two well-defined cannabinoid (CB) receptors. CB1 is a seven transmembrane spanning G-protein-coupled receptor acting by inhibiting cyclic adenosine monophosphate (AMP) release. This receptor is assumed to be the primary neuromodulatory receptor accounting for psychopharmacological effects of THC and most of its analgesic effects [12]. The second receptor, CB2, is a peripheral immunomodulatory receptor, which is also claimed to have important effects on pain. Activation of CB2 suppresses neuropathic pain mechanisms through nonneuronal (i.e., microglia and astrocytes) and neuronal mechanisms that may involve interferon-gamma [13]. THC, considered the classic prototype of cannabinoid, is a weak partial agonist at both CB1 and CB2 receptors [14].

## Anatomical Localization of the Endocannabinoid System Throughout the Pain Pathway

The endocannabinoid system is expressed throughout the ascending and descending pain pathways at peripheral, spinal, and supraspinal sites. CB1 receptors (CB1R) are found on peripheral endings and central terminals of primary afferent neurons. CB1R localization is predominantly presynaptic, directly activated by synthetic agonists, or by endocannabinoids that signal retrogradely, inhibiting the release of neurotransmitters including GABA and glutamate.

The clinical utility of cannabinoids acting at CB1R can be limited due to adverse central side effects and the development of tolerance [15]. This has led to increased interest in the role of the CB2 receptors (CB2R) in pain. More recent evidence in rat models suggests that CB2R is present in the brain under normal and, in particular, under pathological/inflammatory conditions [16], although to a much lesser extent than the ubiquitously expressed CB1R. There is evidence for the expression of CB2R in DRG and in the dorsal horn of the spinal cord and upregulation during neuropathic or inflammatory pain [17]. The high expression of the CB2R in tissues of the immune system including the spleen and thymus as well as on specific immune cells including B lymphocytes, natural killer cells, monocytes, neutrophils, and T lymphocytes has focused research on the viability of the CB2R as a therapeutic target in inflammatory pain conditions in particular, but also neuropathic pain which can have a neuroinflammatory/neuroimmune component [18]. Recent in vivo microdialysis experiments demonstrated that intraplantar injection of the chemical irritant formalin evokes the release of AEA in the midbrain periaqueductal gray (PAG) [19]. The endocannabinoids and N-acylethanolamines also have affinity for, and activity at, a number of non-CB1/non-CB2 receptors, including TRPV1, GPR55 (putative CB3 receptor), and the peroxisome proliferator-activated receptors (PPARs)—all of which are also expressed throughout the pain pathways and likely play important roles in endocannabinoid-mediated regulation of pain.

## Pharmacology of the Cannabinoids

Phyto-, endo-, and synthetic cannabinoids hold distinct and often dose-dependent pharmacological activity at cannabinoid and non-CB1/CB2 receptors, such as members of the transient receptor potential (TRP) family, gamma–aminobutyric acid (GABA) receptors, and serotonin receptors. Cannabinoids can exhibit a partial, full, neutral, and inverse agonists, and antagonist role. When analyzing the two main endogenous cannabinoids, AEA has a high binding affinity to the CB1 receptor but is a partial agonist and thus does not elicit strong intracellular signaling activity. In contrast, 2-AG has a lower binding affinity but elicits a strong intracellular response because it is a full agonist at CB1 [12]. It is shown that 2-AG is more engaged in activity-dependent regulation of synaptic transmission, whereas AEA is involved in more tonic regulation of neuronal activity. Synthetic cannabinoid receptor agonists (SCRAs) referred to as “K2” or “spice” known as new psychoactive substances are examples of highly potent, full agonists at the CB1 receptor. This property partly explains the significant toxicity or death that can result from SCRA use compared to THC.

Each particular cannabinoid has a wide variety of targets through which it can trigger a complexity of responses. Considering the crude cannabis preparations and depending on the plant cultivar and formulation, ratios of cannabinoids may have considerable variations within the plant. The chemical heterogeneity of the cannabis plant constituents extends to terpenes and flavonoids, which hold their own distinct mechanisms of action and pharmacological profiles. It has been advanced that there may be synergy among some constituents of the plant in what is broadly referred to as an “entourage” effect, a concept which will be later described [19]. 

## Analgesic Effects of Cannabis and Mechanism of Action

Preclinical studies using site-specific injections of drugs and conditional knock-out mouse studies have revealed that peripheral, spinal, and supraspinal sites are involved in cannabinoid-mediated analgesia [20••]. A recent meta-analysis of animal studies concluded that CB1 and CB2 receptor agonists, including THC, consistently decreased pain behaviors in inflammatory and nerve-injury models, while CBD and inhibitors of fatty acid amide hydrolase (FAAH) reduced nerve-injury-induced pain behaviors [21]. There are significant limitations with these animal studies due to the risk of bias, since most (> 80%) utilized males even though there is documented sexual dimorphism in cannabinoid pharmacokinetics and behavioral effects [22]. These studies also did not account for the potential influence of side effects, such as motor impairment, catalepsy, or anxiolysis on the measured outcomes (i.e., evoked paw withdrawal to a stimulus). Besides this, in animal models, the concept of multidimensional pain cannot be properly replicated, and there is indeed discordance between the apparent robust effect of cannabinoids in animal models compared to mixed evidence from human studies of cannabis [2]. Also, many published studies on cannabis’ effects are primarily either retrospective studies, not adequately controlled, or of a small sample size. Unless the cannabis was procured from a source that performs quality control on its plants and products to ensure phytocannabinoid content, dosing in clinical populations is an issue and equipotency cannot be applied [20••].

It is known that eCB levels and CB receptor expression are upregulated in local tissues in response to injuries or disease and these are understood to contribute to analgesia [50, 51]. The functional roles of these dynamic changes to the “endocannabinoidome” are not well understood. It is presumed that these changes are protective for the individual.

In clinical studies, total knee arthroplasty patients who developed higher postoperative pain scores (NRS ≥ 5) had significantly increased preoperative levels of the eCB 2-AG in their cerebral spinal fluid and synovial fluid compared to those who reported lower pain levels. Higher synovial fluid 2-AG levels were also correlated with postoperative opioid use [23]. 2-AG is a precursor to proinflammatory eicosanoids [24], and increased levels of 2-AG at baseline may suggest an increased inflammatory burden manifesting as higher pain levels. Patients with fibromyalgia have high levels of circulating eCBs, but their roles are not fully understood in this pathology [25]. Significant decreased levels of salivary 2-AG were identified in patients with trigeminal neuralgia and tension-type headache, while patients with burning mouth syndrome had significantly elevated AEA levels compared to pain-free control patients [20••]. Genetic polymorphisms are correlated with the state of the eCB system and have been associated with pain intensity, opioid-related side effects, and adverse outcomes [19]. On a subjective level, preoperative pressure pain thresholds have been shown to be predictive in various surgical populations [26, 27].

## Therapeutic Pharmaceutical Agents

There are three separated subspecies of cannabis: sativa, indica, and ruderalis. Sativa is the most common strain for all uses. The identification of the chemical composition of cannabis and the possibility of obtaining its pure constituents increased the interest of the scientific community during the last decades. Cannabis plant contains > 500 different compounds, and approximately 125 have been identified and characterized as cannabinoids. These are denominated exogenous cannabinoids or phytocannabinoids. The other compounds are mainly terpenes, flavonoids, and alkaloids. The list of top phytocannabinoids is fairly lengthy. We have ranked the top five and explained briefly their action.

### Δ9-Tetrahydrocannabinol (THC)

Δ9-THC is the major psychotropic component of cannabis being also the most important pharmacological compound [28]. Δ9-THC mediates its biological effect through the activation of the CB1 receptor and is responsible for the psychoactive effects of Δ9-THC, hypolocomotion, hypothermia, catalepsy, and of course, analgesia. Apart from this action, the activation of CB2 has a neuroprotective, antispasmodic, and anti-inflammatory activity [29].

### Cannabidiol (CBD)

CBD is a non-psychoactive phytocannabinoid and is the second most important compound of cannabis from the pharmacological point of view. CBD was discovered to affect the immune function, and it has low affinity to CB1 and CB2, where it can act as a negative allosteric modulator. CBD may produce anti-inflammatory, anti-convulsant, anxiolytic, and neuroprotective effects in a CB receptor-independent manner [30].

### Cannabigerol (CBG)

CBG is another non-psychoactive phytocannabinoid with low affinity for CB1 and CB2 receptors. CBG also inhibits AEA uptake, affecting the ECS. The activity of CBG seems to activate the α2-adrenergic receptor while moderately blocking the serotine 5HT1A receptor. This compound has demonstrated antiproliferative, antibacterial, and anti-glaucoma properties. This compound is studied for possible application in prostate cancer, detrusor overactivity, and bladder pain [29].

### Cannabichromene (CBC)

CBC, the most abundant phytocannabinoid in the plants, is known also to have a low affinity for CB1 and CB2 receptors, affecting the ECS by inhibiting AEA uptake. It is shown to have a strong anti-inflammatory effect in animal models of edema through non-CB receptor mechanisms. In preclinical studies, CBC demonstrated its action and can relieve pain, potentiate the analgesic effects of Δ9-THC, and ameliorate-induced colonic inflammation by inhibiting macrophage and monoacylglycerol lipase (MAGL) activity [29, 30].

### Cannabidivarin (CBDV)

Cannabidivarin, also known as cannabidivarol, is another non-psychoactive cannabinoid compound which has been proven to have anti-epileptic activity [30].

### Cannabis-Derived and Synthetic Cannabis-Related Drugs

Synthetic cannabinoids, particularly agonists of cannabinoid receptors, are more potent than natural cannabinoids. There is one cannabis-derived drug and three synthetic cannabis-related drug products that have been approved for pain management. These include Sativex® (nabiximols) [31], an extract of both THC and CBD not approved in the United States, but approved in Europe and Canada; Epidiolex® [32], a CBD-only extract; and Marinol® [33] and Syndros® [34], which are isomeric, both synthetic forms of THC named dronabinol. Cesamet® [35] (generic name, nabilone) is the only known synthetic cannabinoid approved as a prescription product. These products are labeled for various uses, including cancer-related pain but also other relevant conditions, such as nausea and vomiting associated with chemotherapy, multiple sclerosis pain, severe seizures, and anorexia.

### The “Entourage Effect”

The “entourage effect” is referred to a potentiation of the biological effect of a molecule by related but inactive molecules, acting in combination [29]. The endocannabinoid system demonstrated an “entourage effect” in which several inactive compounds can impact the activity of the primary endocannabinoids, AEA and 2-AG. The interactions between various cannabinoids or between cannabinoids and terpenes are denominated as intra-entourage and inter-entourage, respectively. Applying the “entourage effect,” there is a maximization of the cannabinoid therapeutic efficacy while reducing the adverse side effects [36]. It is the inter-entourage effect that explains the preference of some patients for cannabinoid extracts rather than pure Δ9-THC [29]. The entourage concept has been exploited medicinally in the development of a pharmaceutical drug, nabiximols, which has equal ratios of THC and CBD.

## Evidence from Preclinical Studies

For more than 50 years, cannabinoids have been studied for their analgesic effect in animal models. In 1973, one of the first studies [37] to assess the analgesic effects of THC in mice and rats concluded that cannabinoids hold a greater analgesic activity than aspirin. Besides this fact, the anti-inflammatory action of the synthetic cannabinoid nabilone in a model of acute inflammation was evaluated in rats showing that nabilone action is mediated by an uncharacterized CB2-like cannabinoid receptor [38]. More recently, the effect of cannabidiolic acid (CBDA) and Δ9-THC was assessed in rodents and showed that the CB1 cannabinoid receptor antagonist blocked the anti-hyperalgesia effects of THC, while CBDA’s effects were blocked by a competitive and selective vanilloid receptor 1 [39]. These last two studies concluded that THC produced dose-dependent anti-hyperalgesia and anti-inflammatory effects. CBDA counteracts hyperalgesia via vanilloid receptor 1, conversely THC acts on central and peripheral CB1 receptors, as testified by its selective inhibition.

Cannabinoids have also an impact on pain in preclinical models of traumatic spinal cord injury. Spinal cord injury causes neuropathic pain with various mechanisms, one of which empowers the oxidative stress [28]. It has been shown that cannabinoids suppress behavioral reactions in inflammatory and nerve-injury models, as well as act on pain by mechanical, chemical, and thermal stimuli [40]. Their efficacy and potency may be similar to opioids, and they may even surpass them in neuropathic pain models [41].

## Evidence of Efficacy and Analgesic Power in Clinical Studies

Few studies have examined CBD analgesic effects when administered without other compounds, and little is known regarding dose-dependent effects in non-cannabis users. Testing different doses of CBD in healthy volunteers, CBD did not elicit consistent dose-dependent analgesia and in fact increased pain on some measures [42]. Another randomized controlled trial (RCT) assessing the pain response in non-cannabis volunteers using cannabidiol in induced acute nociceptive pain showed that there was no difference in pain intensity. A placebo-controlled investigation of the analgesic effects, abuse liability, safety, and tolerability of a range allodynia and hyperalgesia as secondary outcomes (CANAB I) [43]. As a follow-up to this study (CANAB II) [44], the effect of single, high-dose oral CBD (1600 mg) was assessed in 24 healthy volunteers in a model of opioid-induced hyperalgesia [145,146]. CBD had no effect on remifentanil-induced hyperalgesia, allodynia, or pain compared to placebo.

Interestingly, there is some observational data including the psychological component of the patient who is using medical cannabis. A study of healthy volunteers assessing the effects of CBD (50 mg) and the human expectation component assigned randomly subjects to various conditions: control group (told they were receiving placebo and received placebo), expectancy group (told they were receiving CBD and given placebo), drug group (told they were receiving placebo and given CBD), and expectancy and drug condition group (told they were receiving CBD and received CBD). Using mimicking pain modulation and offset analgesia, there were no differences reported in pain threshold, intensity, and tolerance [45].

Finally, two systematic reviews and meta-analyses have been published in acute and chronic pain [46, 47]. A lack of benefit in the use of cannabinoids for the management of chronic oncologic and non-oncologic pain was evidenced, either by inconsistent results in pain reduction or by lack of significant impact on physical and emotional functioning. Due to methodological limitations, the latest systematic review conclusions are leading towards “probably beneficial” or “unclear” [48] benefits. Also, some guidelines advocate that cannabinoids are to be considered for pain management as adjuvants or even as substitutes for some therapies. On top of this, a systematic review of randomized controlled trials reinforced the conclusion that most of the available studies are not of sufficient quality to support definitive decision-making, and it is not possible to validate or disprove the medium and long-term efficacy and safety of medical cannabis in pain management [49].

## Evidence in Acute and Postoperative Pain

There have been several studies of CBD in acute pain conditions, but only one to date has shown a signal for analgesic efficacy. A multicenter RCT, enrolling 99 patients concluded that buccally absorbed CBD demonstrated an acceptable safety profile and showed significant promise in the reduction of pain in the immediate perioperative period after arthroscopic rotator cuff repair compared with the control [50].

The topical use of CBD was studied in acute postoperative pain; however, the local effects of topical CBD as a supplement to multimodal analgesia were not beneficial for providing additional pain relief. Some limitations of this study were that the CBD used was an over-the-counter product, its skin penetrance was not assessed, and local and systemic levels of CBD and its metabolites were not measured [51]. Also, another RCT of patients undergoing ureteroscopy where the study group received 20 mg cannabidiol oil daily for 3 days postoperatively found that cannabidiol oil is safe but ineffective when compared to placebo in reducing post-ureteroscopy stent discomfort or opioid usage. Dizziness was more common in the CBD group and other adverse effects included drowsiness, tiredness, constipation, and poor sleep [52].

Between 2016 and 2019, clinical outcomes of patients using medical cannabis for postoperative pain have been evaluated in the US, suggesting that the role of medical cannabis may be limited due to the non-significant clinical benefits in pain control. It showed that CBD use was associated with a possible increased risk of side effects, such as postoperative hypotension [53]. However, all human studies may be underpowered with small sample sizes.

### Musculoskeletal Pain

The evidence backing the use of MC in musculoskeletal pain is also scarce and there are only a few studies targeting low back pain. CBD is recommended for musculoskeletal pain with failure or intolerance to first or second-line treatments [54]. In a double-blind RCT on 100 patients with acute low back pain, the use of CBD showed no superiority of the drug over placebo [55]. However, the available evidence is not of good enough quality to make a recommendation in musculoskeletal pain [56].

The CANBACK trial investigated the analgesic efficacy and safety of single dose of 400 mg oral CBD as an adjunct to standard care with those of placebo in patients presenting to an emergency department with acute non-traumatic low back pain. The study found no difference between the CBD and placebo groups with respect to the primary outcome (pain scores 2 h after administration). Furthermore, there were no differences in secondary outcomes; median lengths of staying were similar; acetaminophen and opioid use during the 4 h before and the 4 h after receiving CBD or placebo was similar for the two groups, as was the incidence of side effects [54]. The CANBACK trial is the largest RCT that studied cannabinoids in acute low back pain. In this study, as in many others, the 400 mg dose selected was based on safety and toxicology data for humans [57], and on prior CBD studies assessing its therapeutic properties, suggesting that peak blood concentration was reached at 1–2 h and that it was appropriate use for the emergency department care.

In a systematic review and meta-analysis comparing cannabinoids versus placebo in all types of pain, cannabinoids had no effects on improving acute pain and had an increased risk of non-serious adverse events. It appears that in some cases, the undesirable effects of cannabinoids used for pain may outweigh the potential benefits [58]. The over-the-counter availability of CBD in certain countries will make it difficult to restrict its use for treating acute low back pain, even though it is now known to be ineffective for this condition.

## Cannabis and Cannabinoids in the Perioperative Period

CBD is widely marketed and available in the United States and many other countries, and an increasing number of people are using it for recreational reasons and to treat a variety of conditions, where approximately 62% reported using CBD for a medical purpose, mostly for chronic pain [59]. Another study describing the demographics of CBD users found that the majority substituted CBD for other drugs, including opioids (53%), non-steroidal anti-inflammatory drugs, gabapentinoids, or benzodiazepines [60].

CBD use is common in patients presenting for orthopedic surgical care and particularly in patients presenting with low back pain, with 25% of respondents using CBD [61], while other reports showed 22% of patients using CBD during the postoperative period in total hip or knee replacement surgery [62]. Similarly, there were no reported differences in postoperative pain level between those who used CBD and those who did not. A total of 19% of patients who were assessed in an orthopedic sports medicine clinic reported using CBD, and 31% of them also reported using marijuana. In contrast, the pain level in the affected joint was significantly higher in CBD users [63].

Clinicians may consider delaying elective surgery in situations of acute intoxication. Intraoperatively, patients with recent cannabis use may have increased anesthetic requirements. Cannabis could have interactions with other medicines and could increase or depress their effects, as well as the metabolism of certain drugs. In the postoperative phase, habitual cannabis users may have difficulties in pain control and higher opioid needs [64]. Habitual cannabis patients are exposed to other potential challenges postoperatively, including complications related to respiratory, cardiovascular, neurologic, and renal systems, as well as risks of cannabis withdrawal. These patients are exposed to other longer-term complications such as worse pain, functional impairment, mood disturbance, and hospital readmission [65].

Care providers should inform and discuss potential risks before surgery and address relevant concerns and make an appropriate postoperative care and follow-up [65, 66].

## Conclusions

The basic science of cannabis is extensive, but it does not translate into relevant clinical outcomes in human research. Dosing is often difficult for cannabinoid trials, as dose–response studies have not been undertaken. The use of cannabinoids in acute pain did not yield statistically significant results in improving acute pain. Large randomized, controlled trials evaluating diverse cannabis extracts are needed in different clinical pain populations to determine safety and efficacy.

## Data Availability

No datasets were generated or analysed during the current study.
